# Atypical chest radiological features in Covid 19: Case based review

**DOI:** 10.1186/s43055-022-00729-9

**Published:** 2022-03-01

**Authors:** M. Vishnu Sharma, N. Anupama

**Affiliations:** 1 Department of Respiratory Medicine, A J Institute of Medical Sciences & Research Centre, Kuntikana, Mangalore, Karnataka India; 2 Department of Physiology, Kasturba Medical College, Mangalore, Manipal Academy of Higher Education, Manipal, India

**Keywords:** Atypical radiological features in COVID-19, Chest imaging in COVID-19, Bronchopneumonia in COVID-19, Complications of COVID-19

## Abstract

Chest imaging plays an important role in the diagnosis and management of patients with COVID-19. Some patients may have atypical lesions on chest image. Awareness about the atypical imaging features is essential to avoid misdiagnosis/delayed diagnosis. Atypical chest imaging features in COVID-19 include central involvement, peribronchovascular involvement, isolated upper lobe involvement, nodular involvement, lobar consolidation, solitary involvement, unilateral lung involvement, interstitial emphysema, pneumomediastinum, subcutaneous emphysema, pneumothorax, hydropneumothorax, mediastinal adenopathy, cavitory lesions, bulls eye sign, necrotizing pneumonia with abscess, empyema, pleural and pericardial effusion, and subpleural sparing. In patients with atypical chest imaging features, when RT-PCR test results are positive diagnosis is certain. Diagnostic difficulty may arise when RT-PCR test results are negative. In such cases a proper epidemiologic history, typical clinical features, and exclusion of other causes for a similar chest imaging features may help in diagnosis. Causes for atypical chest imaging features include early stage of the disease when lesion can be unilateral or focal or single, late stage of the disease when lesions regress, coexisting diseases/conditions, preexisting lung parenchymal diseases, fluid overload, complications like other bacterial/ fungal infection/tuberculosis/barotrauma or involvement of other organs like kidney, heart, or liver which may lead to pleural effusion. Iatrogenic trauma, barotrauma, or drug-induced immunosuppression leading to opportunistic infections can also lead to chest imaging features. Some of the CT features like cavitory lesion, mediastinal adenopathy, and pleural and pericardial effusion may be due to complications during the course of the disease or coexistent diseases. In this pictorial essay we discuss some atypical chest images with salient learning points from each case. Awareness about the atypical chest imaging features is essential to avoid misdiagnosis/delayed diagnosis. Some of the atypical features may require further evaluation/follow up and management.

## Background

Chest imaging plays an important role in diagnosis and management of patients with COVID-19. The majority of patients with COVID-19 bronchopneumonia have typical chest imaging features—bilateral, basal, peripheral, and subpleural bronchopneumonia [1]. Ground glass opacities and consolidation are the most common initial radiological findings. Typical chest imaging features help to suggest the diagnosis in the appropriate setting. It should be remembered that chest imaging is not the standard for the diagnosis of COVID-19 as many other diseases can mimic COVID-19 in chest imaging and vice versa. Chest imaging findings should be correlated with epidemiologic history, clinical presentation, and reverse transcription polymerase chain reaction (RT-PCR) test results [1]. Chest imaging has a low positive predictive value (1.5% to 30.7%) in low-prevalence regions, and high negative predictive value which ranged from 95.4 to 99.8% for nCovid pneumonia. Pooled sensitivity and specificity for chest CT are 94% to 96% and 37%, respectively, in detecting COVID-19 bronchopneumonia [2].

Some patients with COVID-19 may show atypical chest imaging features. Awareness about these atypical chest imaging features is important to avoid misdiagnosis/delayed diagnosis. When atypical, diagnosis of COVID-19 pneumonia should be by exclusion of other causes for the radiological abnormality along with correlation of epidemiologic history, clinical presentation, and RT-PCR test results [1]. In some cases, atypical chest imaging features may suggest complications, preexisting lung diseases, or comorbidity [3]. Early identification of the cause for atypical chest imaging features may help in appropriate treatment and improve the outcome.

Atypical chest imaging features in COVID-19 described in the literature include central involvement, peribronchovascular involvement, isolated upper lobe involvement, nodular involvement, lobar consolidation, solitary involvement, unilateral lung involvement, interstitial emphysema, pneumomediastinum, subcutaneous emphysema, pneumothorax, hydropneumothorax, mediastinal adenopathy, cavitory lesions, bullseye sign, necrotizing pneumonia with abscess, empyema, pleural and pericardial effusion, and subpleural sparing [3–5].

In patients with atypical chest imaging features, when RT-PCR test results are positive diagnosis is certain. Diagnostic difficulty may arise when RT-PCR test results are negative. In such cases a proper epidemiologic history, typical clinical features, and exclusion of other causes for a similar chest imaging features may help in diagnosis.

Causes for atypical chest imaging features include early stage of the disease when lesion can be unilateral or focal or single, late stage of the disease when lesions regress, coexisting diseases/conditions, preexisting lung parenchymal diseases, fluid overload, complications like other bacterial/fungal infection/tuberculosis/ barotrauma, involvement of other organs like kidney, heart, or liver which may lead to pleural effusion. Iatrogenic trauma, barotrauma, or drug-induced immunosuppression leading to opportunistic infections may also lead to chest imaging features [3–5]. Some of the CT features like cavitory lesion, mediastinal adenopathy, and pleural and pericardial effusion may be due to complications during the course of the disease or coexistent diseases [3]. In this pictorial essay we discuss some atypical chest imaging manifestations.

## Main text

### Case 1

**Fig. 1:**
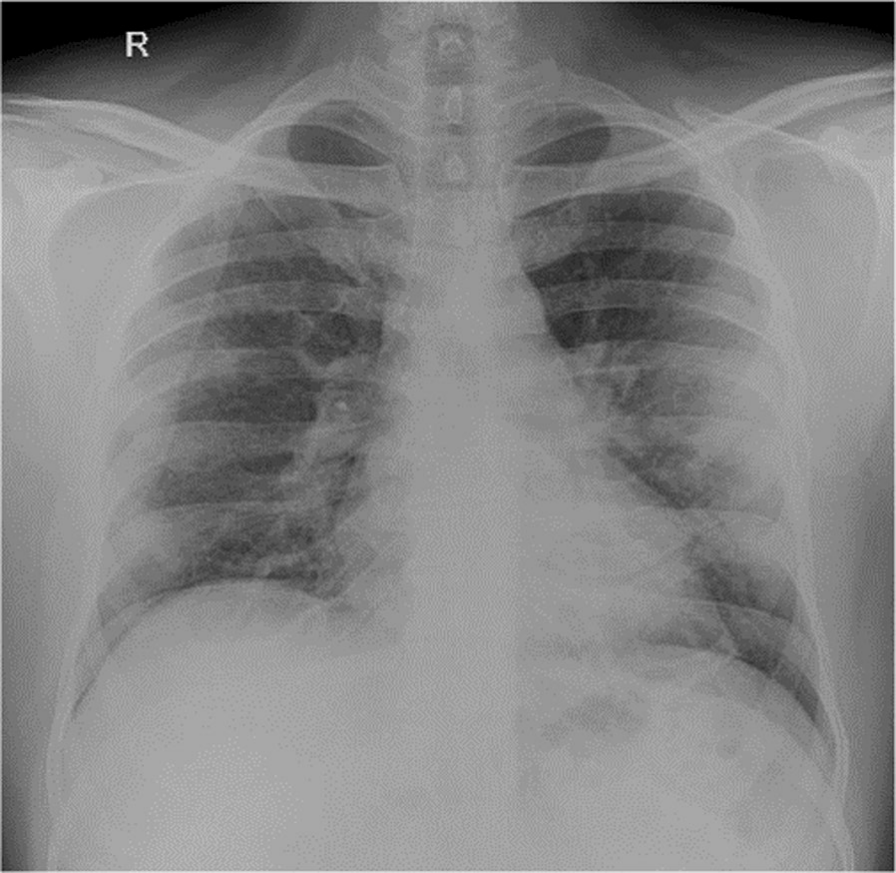
Peripheral opacity predominantly in left mid- and lower zone. Minimal opacity is seen on right mid- and lower zone

A 34-year-old man admitted with typical symptoms suggestive of COVID-19 bronchopneumonia, confirmed by RT-PCR. He had fever, cough, and breathlessness. His oxygen saturation was 94% while breathing room air, on admission. Chest X-ray (Fig. 1) was taken on admission, day 6 after the onset of fever and upper respiratory tract symptoms. X-ray (Fig. 1) shows peripheral opacity predominantly in left mid- and lower zone. Minimal opacity is seen on right mid- and lower zone. Ground glass opacity is the initial radiological abnormality which typically starts in the lower lobes, right more often than left, usually bilateral, peripheral, and subpleural. In some patients with early stage of the disease it may be prominent on one side as in this case.

#### Learning point

In early stage of COVID-19 bronchopneumonia, radiological lesions may be unilateral, or may be more predominant on one side.

### Case 2

**Fig. 2:**
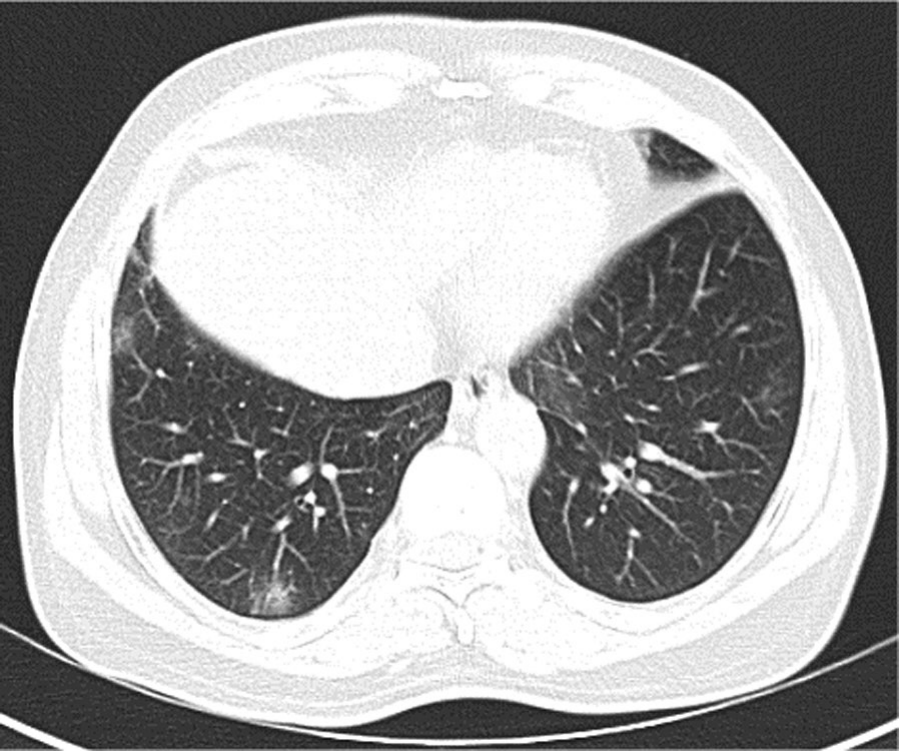
Right lower lobe peripheral ground glass opacity suggestive of early bronchopneumonia with minimal changes of early ground glass opacity in left lower lobe also

A 56-year-old man with uncontrolled diabetes, hypertension, and coronary artery disease on treatment was admitted with fever and upper respiratory symptoms of 5 days’ duration. Chest CT was done as he had developed new onset of cough on the day of admission. CT (Fig. 2) showed right lower lobe peripheral ground glass opacity suggestive of early bronchopneumonia with minimal changes of early ground glass opacity in left lower lobe also. COVID-19 was confirmed by RT-PCR. He made uneventful recovery with treatment.

#### Learning point

Unilateral, lower lobe predominant lesion may indicate early stage of the COVID-19 bronchopneumonia. With treatment these lesions can regress. Bilateral involvement and typical radiographic features may appear in such patients if the disease progresses.

### Case 3

**Fig. 3:**
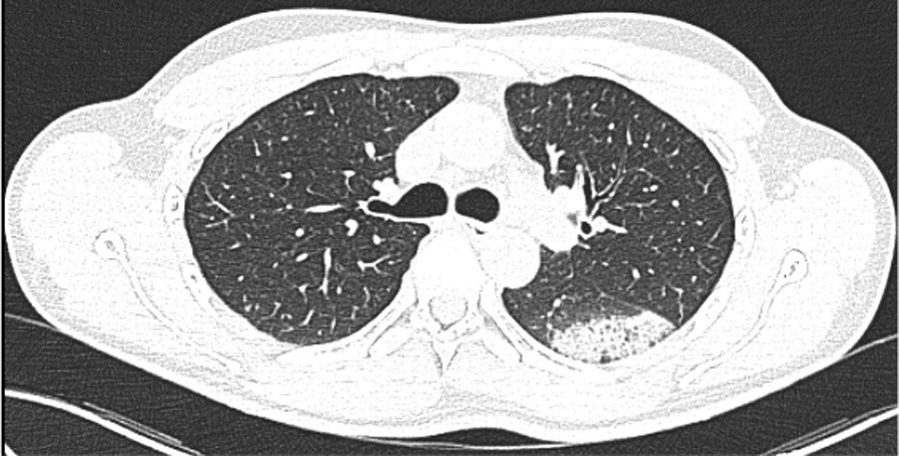
Unilateral peripheral patchy areas of consolidation suggestive of bronchopneumonia in the left lower lobe

**Fig. 4:**
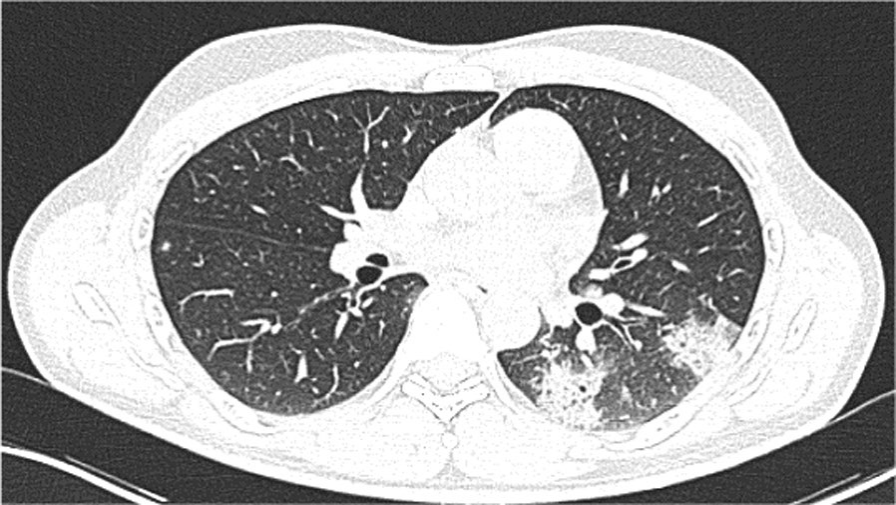
Unilateral peripheral patchy areas of consolidation suggestive of bronchopneumonia in the left lower lobe

**Fig. 5:**
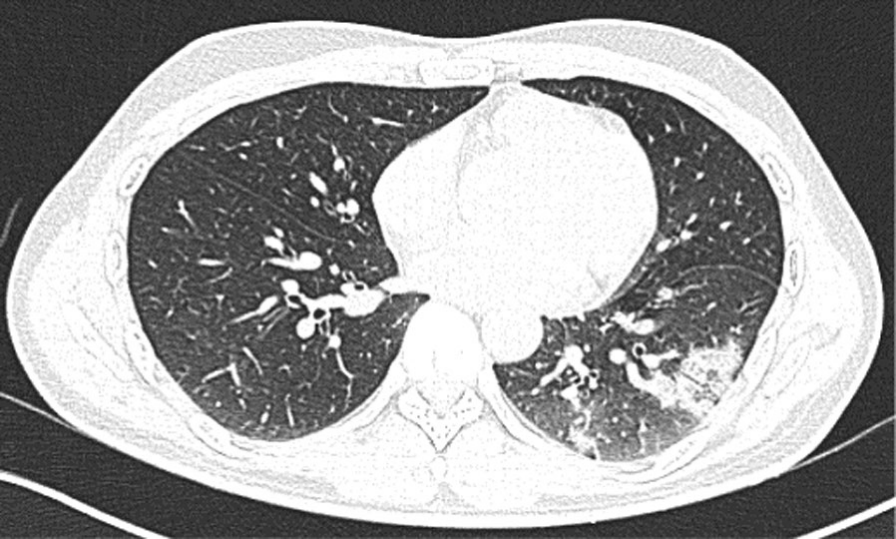
Unilateral peripheral patchy areas of consolidation suggestive of bronchopneumonia in the left lower lobe

A 49-year-old male was admitted with fever and upper respiratory symptoms of 8 days’ duration. Chest CT (Figs. 3, 4, 5) was done as he had developed new onset of cough two days prior to admission. CT showed unilateral peripheral patchy areas of consolidation suggestive of bronchopneumonia in the left lung. COVID-19 was confirmed by RT-PCR. He had no hypoxia or any other complication. He made uneventful recovery with treatment.

#### Learning point

In some patient with mild or moderate COVID-19 bronchopneumonia, lesions may remain unilateral. When lesions remain unilateral and predominantly ground glass opacity is seen, it may indicate milder form of the disease.

### Case 4

**Fig. 6:**
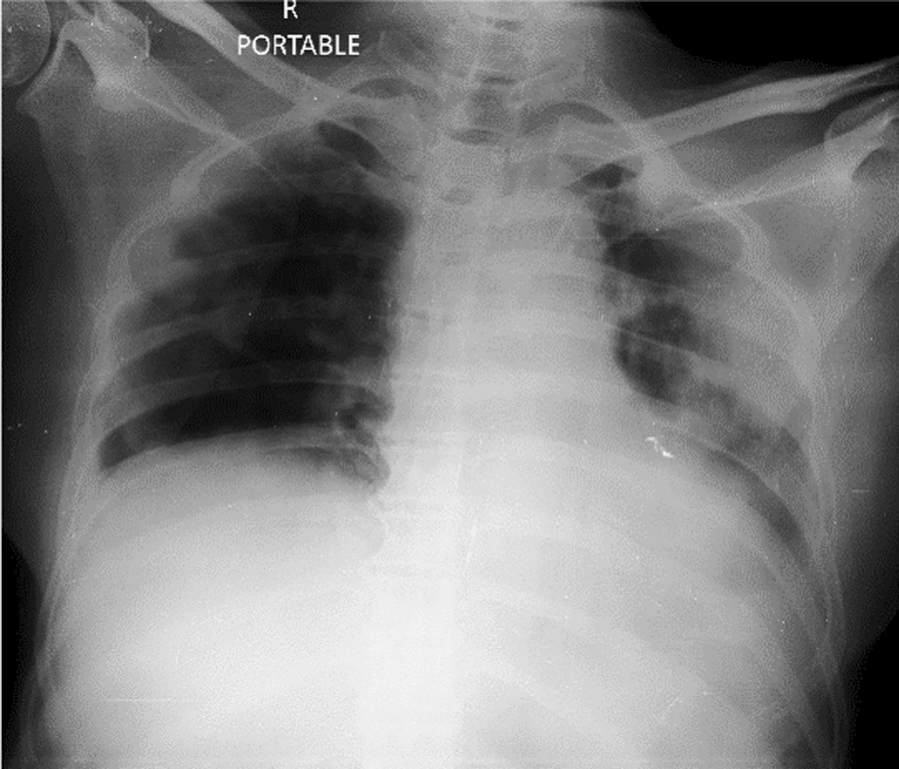
Chest X-ray showing bronchopneumonia in left lung predominantly in the mid-zone

**Fig. 7:**
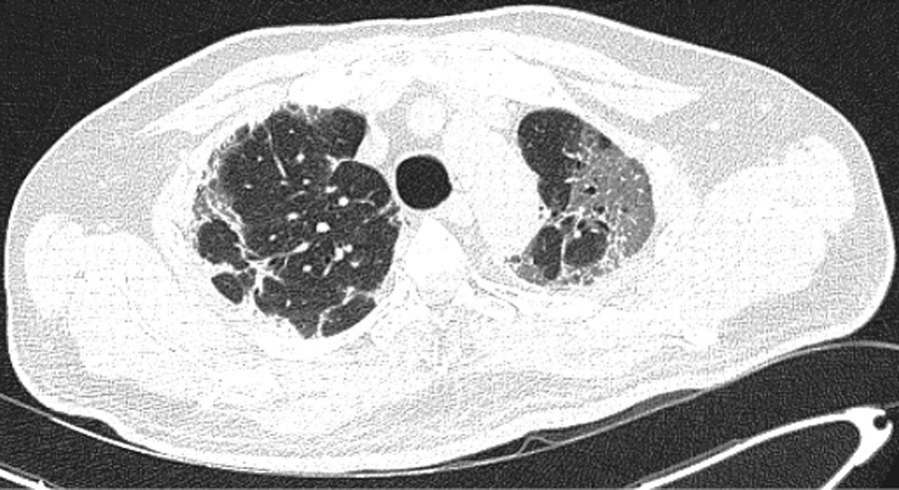
CT shows areas of consolidation and crazy paving in the upper lobes indicating early stage of lung involvement

**Fig. 8:**
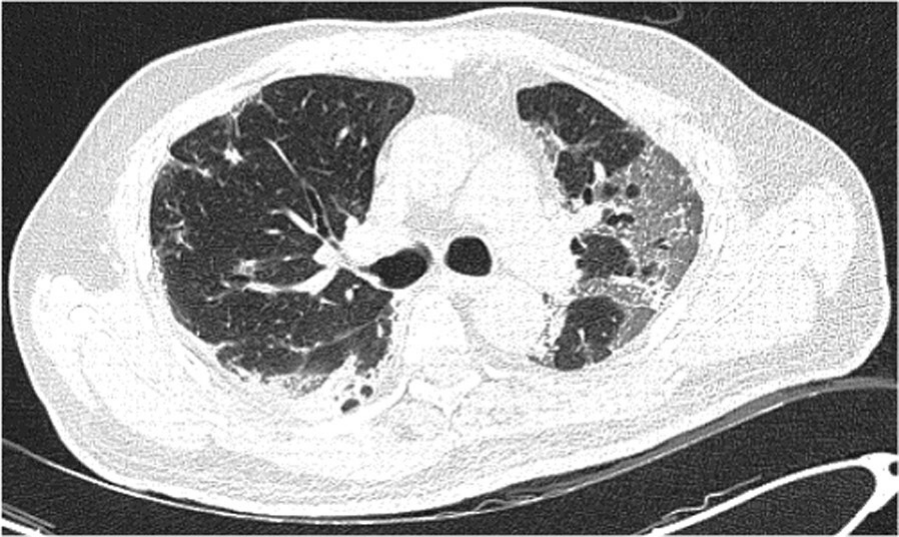
CT shows areas of consolidation and crazy paving in the upper lobes indicating early stage of lung involvement

**Fig. 9:**
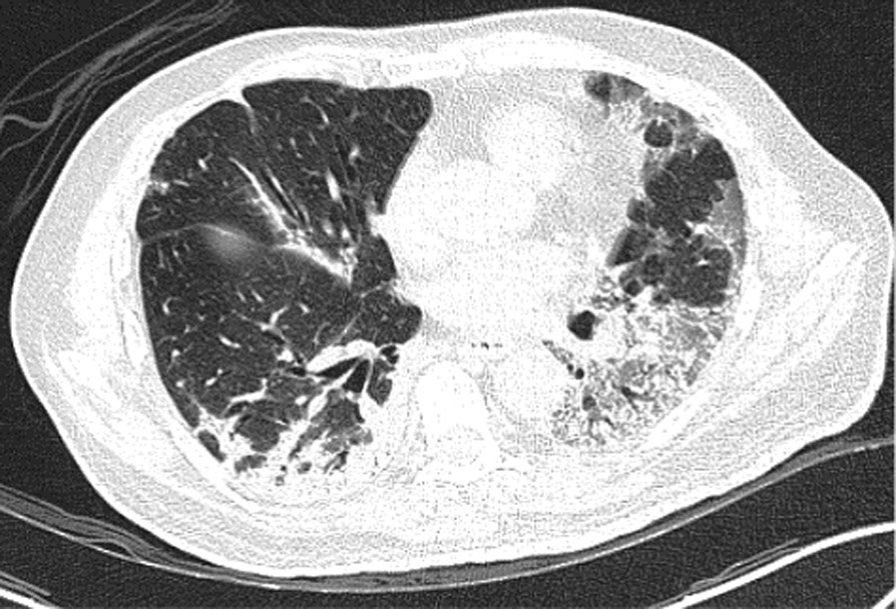
CT shows areas of fibrosis, bands, and dilatation of lower lobe bronchioles on left side indicative of stage of resolution

A 58-year-old male with confirmed COVID-19 was referred from peripheral hospital on day 12 of illness due to progressive hypoxia. Chest X-ray (Fig. 6) and CT scan (Figs. 7, 8, and 9) shows asymmetrical lesions, more on left side. CT shows areas of consolidation and crazy paving in the upper lobes indicating early stage of lung involvement, fibrosis, bands, and dilatation of lower lobe bronchioles on left side indicative of stage of resolution.

#### Learning point

During the stage of resolution lesions can be asymmetrical. Various stages of the lesion may be seen in the same patient in chest imaging when the disease is progressive.

### Case 5

**Fig. 10:**
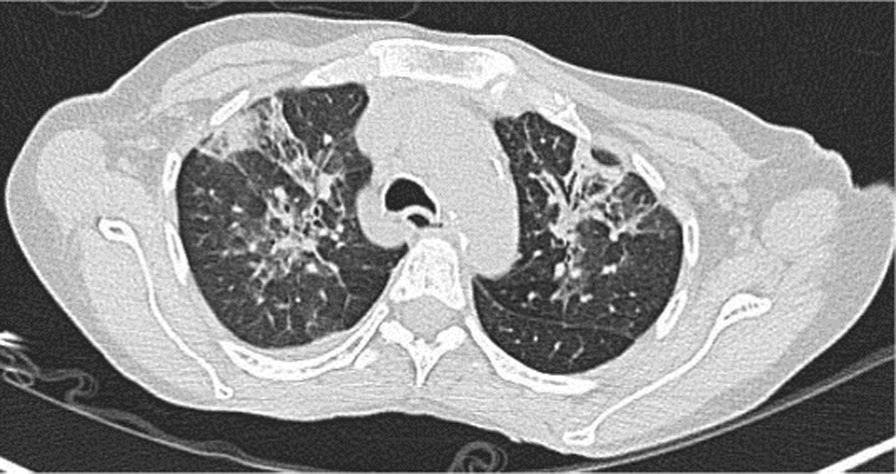
Central and peribronchovascular lesions with organizing pneumonia pattern

**Fig. 11:**
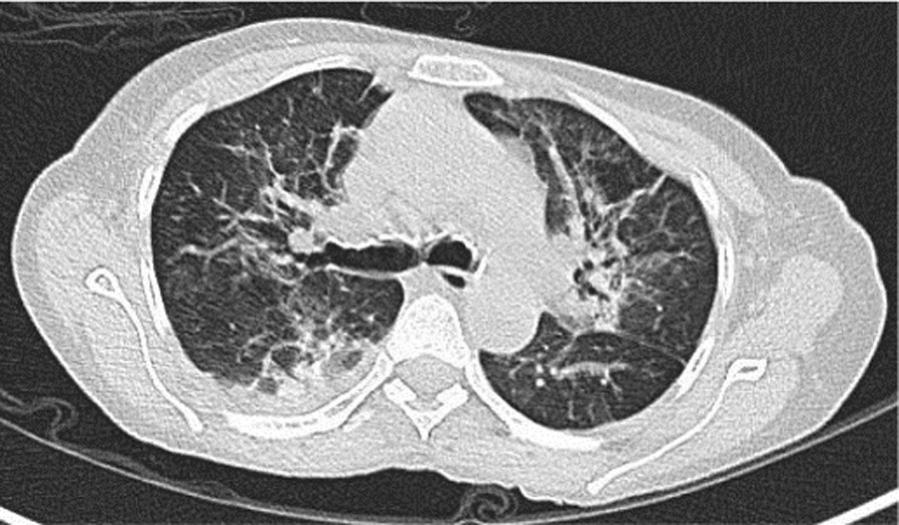
Central and peribronchovascular lesions with organizing pneumonia pattern

**Fig. 12:**
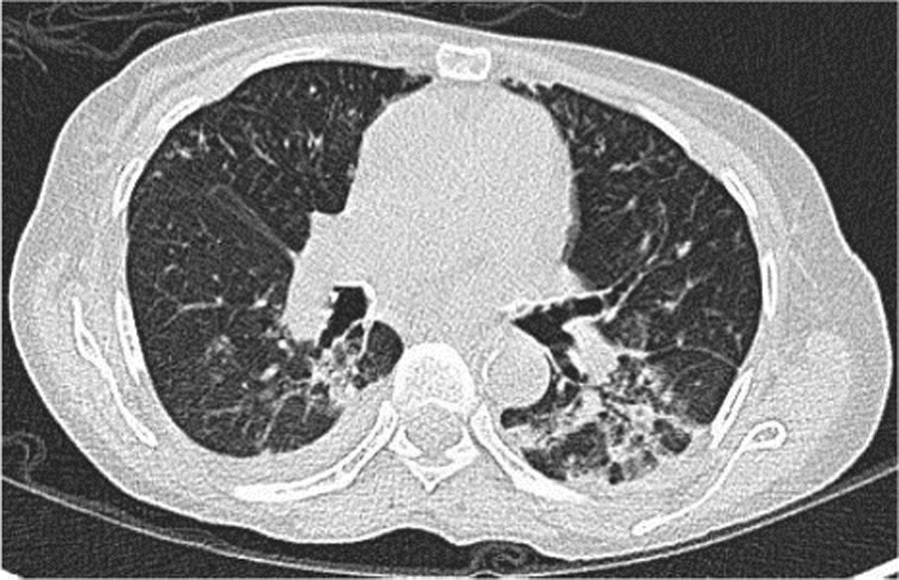
Central and peribronchovascular lesions with organizing pneumonia pattern

**Fig. 13:**
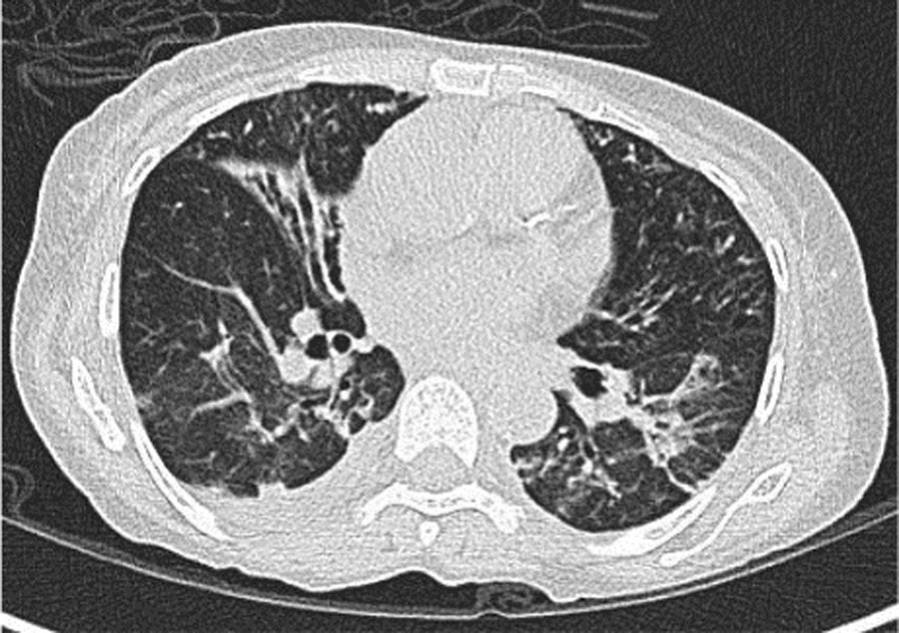
Central and peribronchovascular lesions with organizing pneumonia pattern

**Fig. 14:**
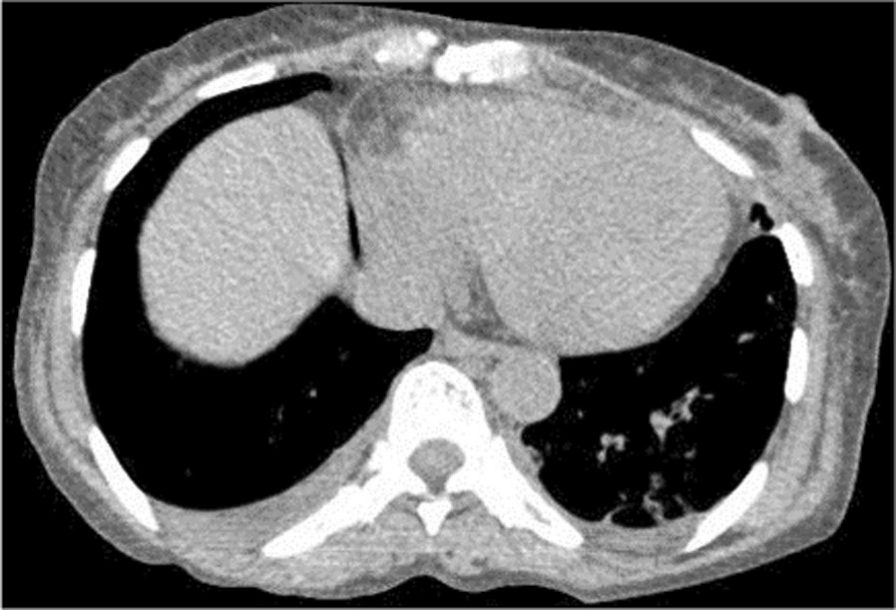
A small pleural effusion on right side

A 57-year-old female diabetic with chronic renal failure on irregular hemodialysis was admitted with increasing breathlessness and cough since 10 days. She had no other symptoms suggestive of COVID-19. On admission it was thought that her symptoms were due to volume overload as she had not undergone dialysis since 2 weeks. As there was no improvement even after two dialysis CT scan thorax was done. RT-PCR confirmed COVID-19. CT (Figs. 10, 11, 12 and 13) shows central and peribronchovascular lesions with organizing pneumonia pattern and a small pleural effusion on right side (Fig. 14). She had no other features of secondary or opportunistic respiratory infection. Sputum examination and bronchoscopic analysis did not yield any pathogen. She made uneventful recovery with dialysis and treatment for COVID-19 bronchopneumonia. Small right-sided pleural effusion was probably due to fluid overload. It disappeared after dialysis.

#### Learning point

In patients with comorbidities and in patients with moderate or severe COVID-19, atypical chest imaging features need further evaluation to find the cause. Other infections, non-infectious diseases, comorbidities, and complications of COVID-19 can lead to atypical radiological features. Identifying the cause is essential for proper treatment to improve the outcome.

### Case 6

**Fig. 15:**
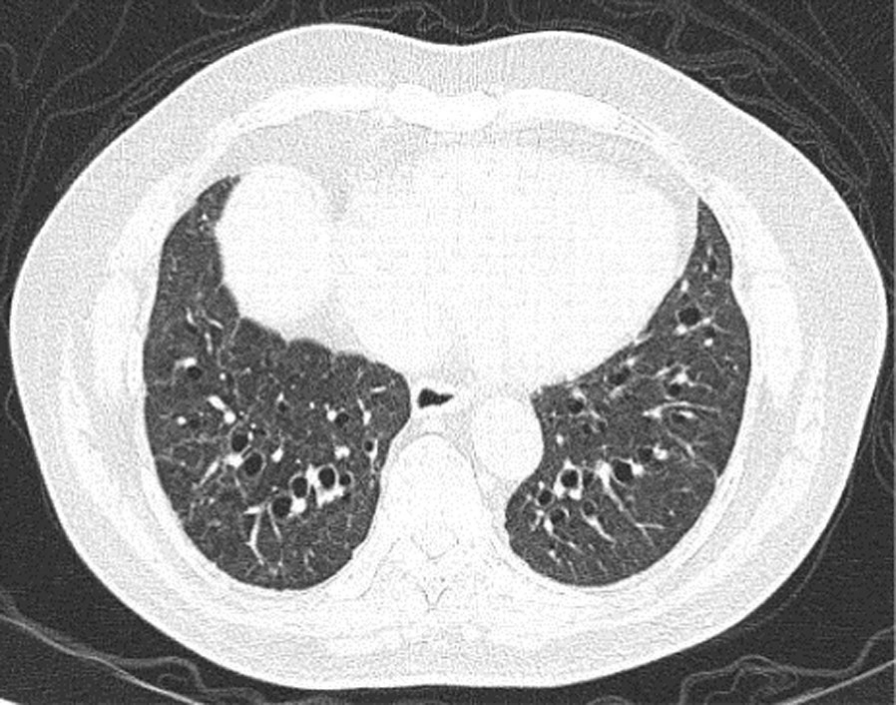
Bilateral lung cysts

A 48-year-old man was admitted for evaluation of long COVID-19. He had COVID-19 6 weeks back but continued to have occasional cough, exertional dyspnea. CT scan (Fig. 15) shows bilateral lung cysts. Lung cysts may be formed following COVID-19 bronchopneumonia due to ischemic parenchymal damage, lung fibrosis, low lung compliance, and inflammatory exudate in the airways [6–8]. These cysts usually appear after 3 weeks during the stage of recovery from lung injury during the process of repair. It may be more common in patients who had moderate or severe disease, those who required oxygen/noninvasive or invasive ventilatory support and prolonged intensive care unit stay [7, 8]. It may lead to post-COVID-19 respiratory insufficiency and dyspnea.

#### Learning point

Lung cysts may occur in some patients following COVID-19 bronchopneumonia. It may be more common in patients who had more severe disease and may lead to persistent respiratory symptoms.

### Case 7

**Fig. 16:**
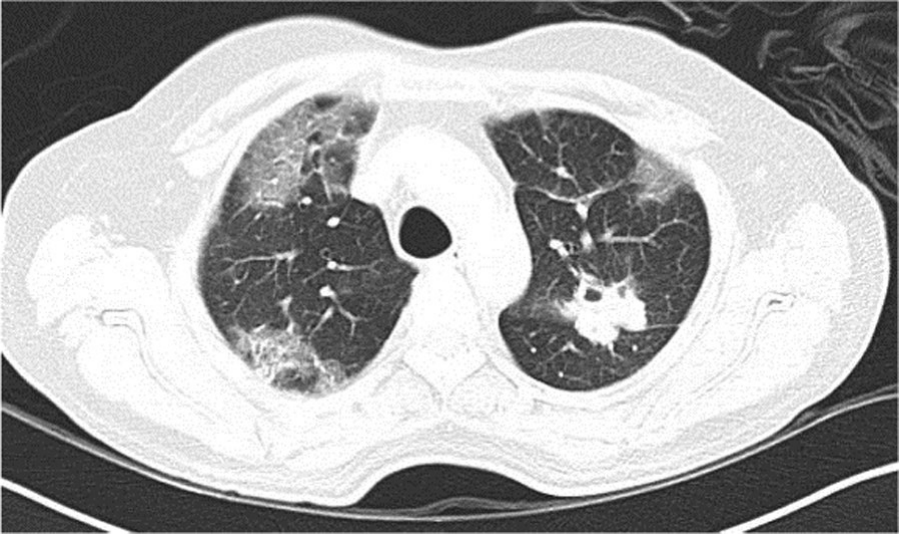
A dense opacity in left upper lobe in addition to bilateral lesions suggestive of COVID-19 bronchopneumonia

A 48-year-old man admitted with confirmed COVID-19. CT scan (Fig. 16) shows a dense opacity in left upper lobe in addition to bilateral lesions suggestive of COVID-19 bronchopneumonia. He was a non-smoker, had no other risk factors for malignancy. He had no preexisting lung disease, had no comorbidity. He had no respiratory symptoms prior to COVID-19. He was advised further evaluation with contrast CT and bronchoscopy 2 weeks after recovery. Contrast CT was suggestive of malignancy. CT guided biopsy confirmed adenocarcinoma.

CT scan thorax may show additional abnormalities in some patients. These lesions should be carefully evaluated and should be correlated clinically. Some of these lesions may be due to preexisting lung diseases, comorbidities, or intrathoracic tumors [9]. Further evaluation and follow-up is essential in such patients where the etiology is not clear. If the lesion is suspicious of malignancy contrast CT scan should be done.

#### Learning point

When CT scan thorax shows additional abnormalities which are not likely to be due to COVID-19, these should be carefully evaluated and should be correlated clinically.

### Case 8

**Fig. 17:**
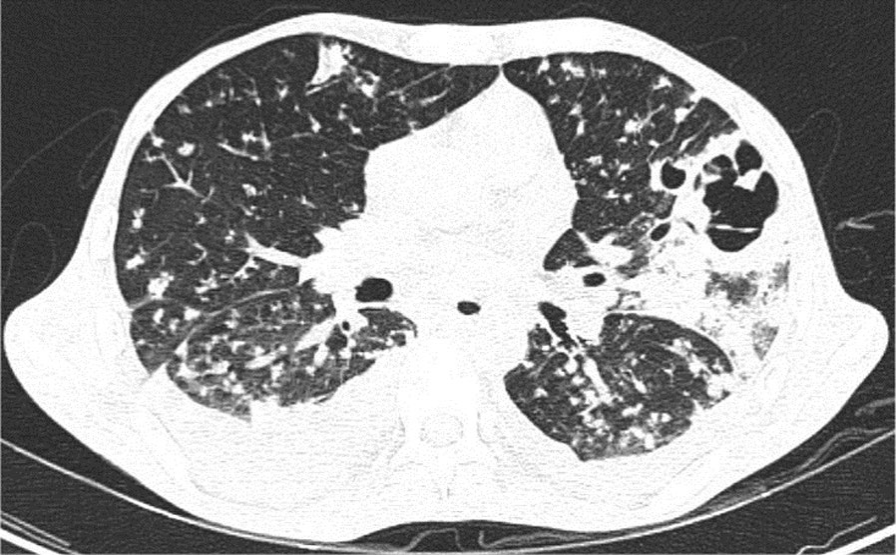
Bilateral infiltrates with tree in bud appearance and cavity in left upper lobe

**Fig. 18:**
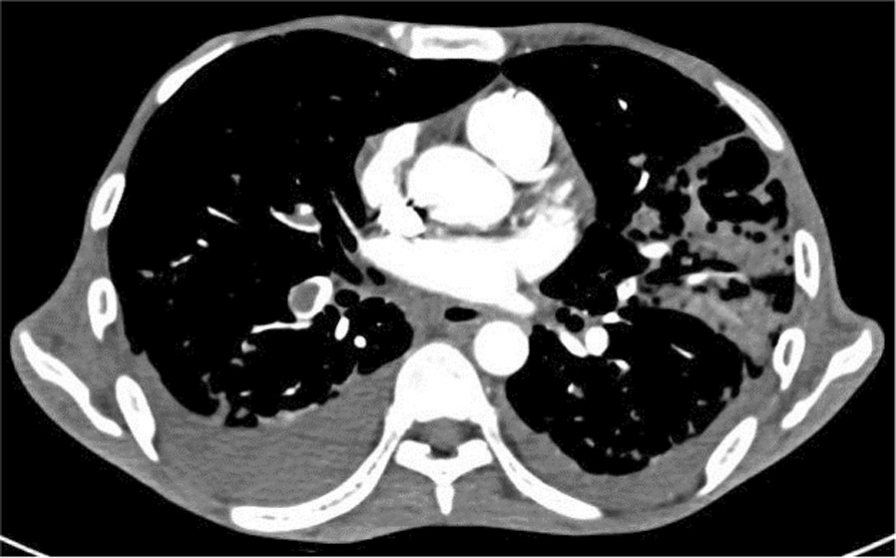
Bilateral small pleural effusion and right-sided pulmonary embolism

A 54-year-old male, uncontrolled diabetes, was admitted after 3 weeks following COVID-19 bronchopneumonia from which he had partially recovered. After an initial improvement, he continued to have low-grade fever, increasing cough, occasional mild hemoptysis, and exertional dyspnea. CT scan (Fig. 17) showed bilateral infiltrates with tree in bud appearance and cavity in left upper lobe. Bilateral small pleural effusion and right-sided pulmonary embolism was also seen (Fig. 18). Sputum smear examination showed Acid fast bacillus, gene x-pert confirmed mycobacterium tuberculosis, rifampicin resistance was not detected. He made uneventful recovery with anti-tuberculosis treatment. Bilateral small pleural effusion was due to malnutrition and hypoprotinemia.

Cavity in lung due to COVID-19 is unusual. Common cause for cavity in the lung in a patient with COVID-19 includes pulmonary tuberculosis, necrotizing pneumonia, fungal infection like mucormycosis, preexisting cavity (most often post-tubercular) [5]. Rarely cavity can occur in COVID-19 without any other obvious cause. Hence, the presence of cavity in lung in COVID-19 requires further evaluation to find the etiology of the cavity. Sputum examination should be done in all such cases. Bronchoscopy may be required if sputum examination is noninformative.

#### Learning point

The presence of cavitating lung lesion in a patient with COVID-19 requires further evaluation as cavity is often due to other causes, infection being the most common.

### Case 9

**Fig. 19:**
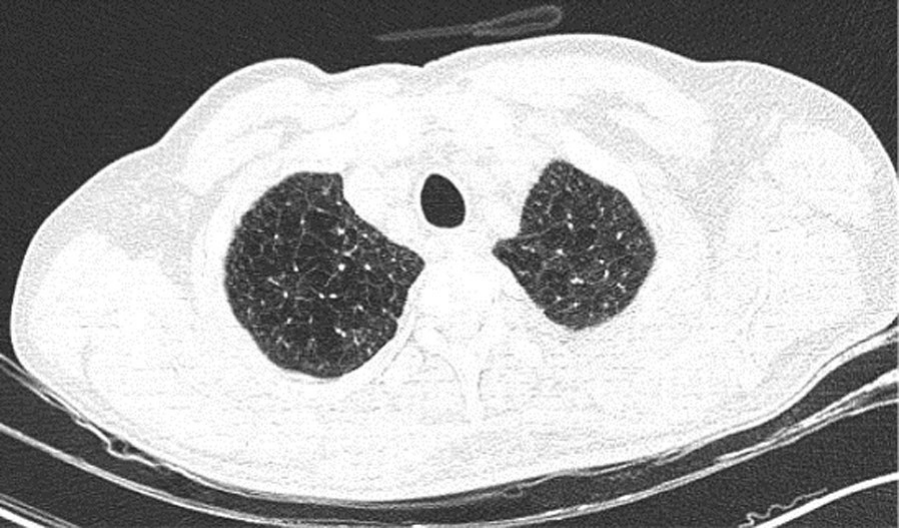
Pan acinar emphysema

**Fig. 20:**
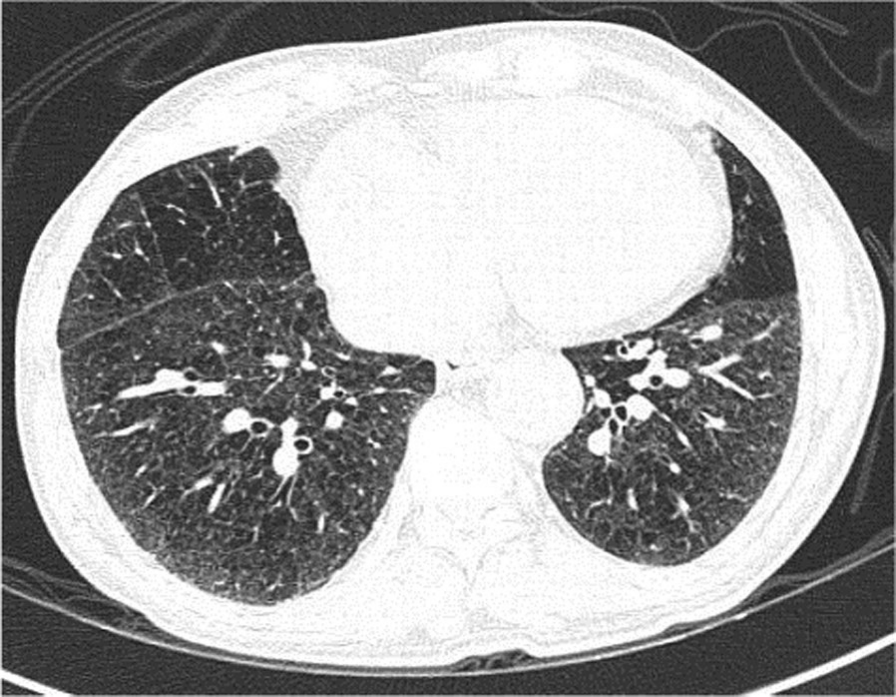
Pan acinar emphysema

**Fig. 21:**
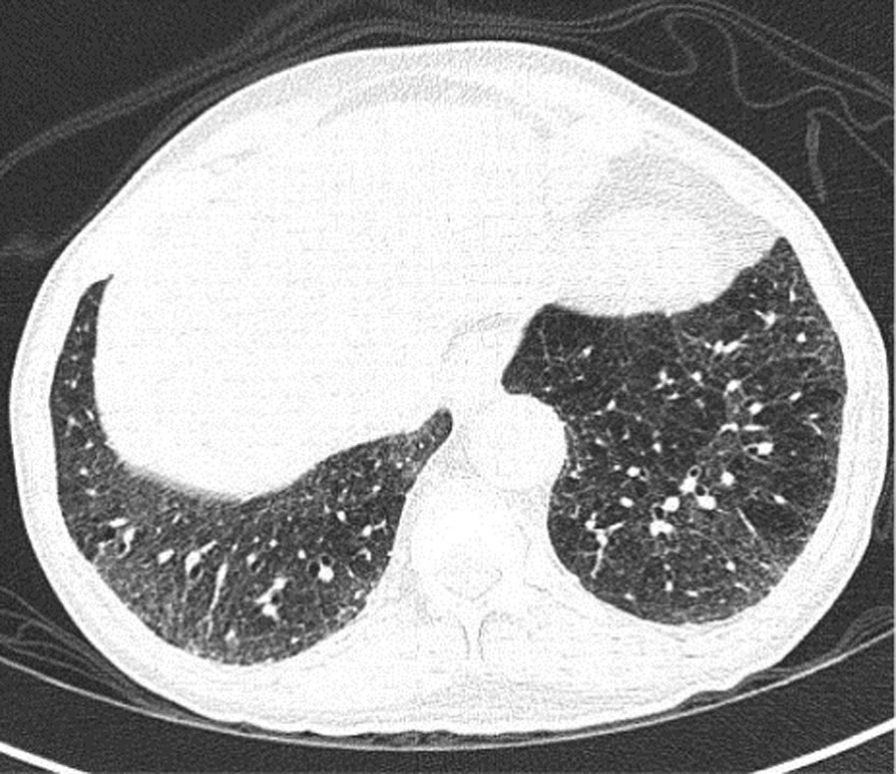
Pan acinar emphysema

A 60-year-old smoker admitted on day 7 following fever and upper respiratory symptoms. COVID-19 was confirmed by RT-PCR. CT scan thorax was done as his room air saturation was 92%. CT scan (Figs. 19, 20 and 21) shows pan acinar emphysema. There was no evidence of COVID-19 bronchopneumonia. His hypoxia was due to COPD, pan acinar emphysema. His hypoxia improved with bronchodilators. Since there was no evidence of lung involvement due to COVID-19, systemic steroid and anticoagulant was not administered. He made uneventful recovery.

Hypoxia in patients with COVID-19 may be due to causes other than bronchopneumonia. Preexisting lung diseases, obstructive airway diseases, cardiac, renal, liver diseases, metabolic disorders, anemia, respiratory muscle dysfunction, and obstructive sleep apnea can lead to hypoxia [10]. These should be diagnosed and treated promptly. Detailed history, focused physical examination, and appropriate investigations are the key to identify the multifactorial causes for hypoxia. Some patients with preexisting lung diseases may have exacerbation of the disease and hypoxia during COVID-19. Identifying the cause for hypoxia helps to plan treatment and avoid unnecessary medications.

#### Learning point

CT scan thorax in patients with COVID-19 should be carefully evaluated for coexisting intrathoracic diseases which can cause/contribute to dyspnea and hypoxia.

### Case 10

**Fig. 22:**
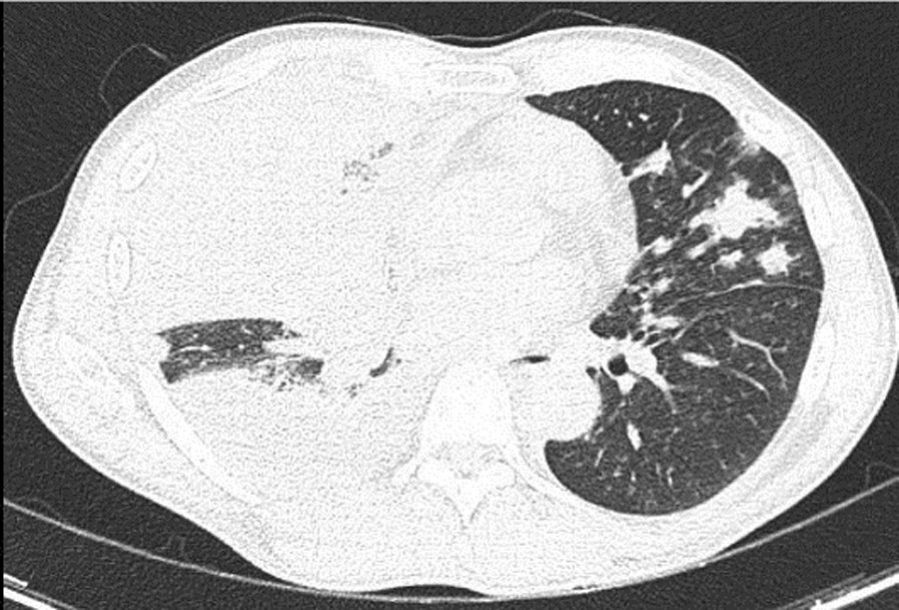
Right-sided pleural effusion

**Fig. 23:**
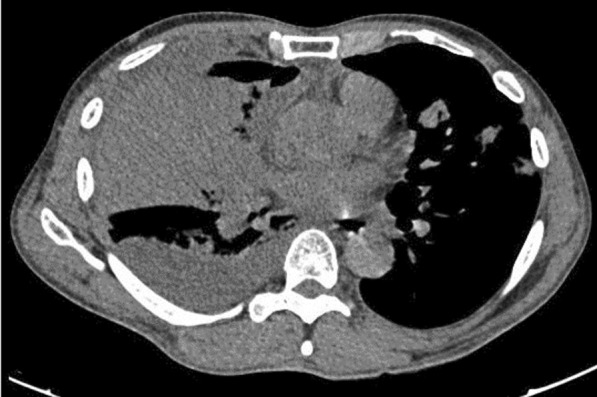
Right-sided pleural effusion

A 48-year-old male with alcoholic cirrhosis liver was admitted with fever, dyspnea. COVID-19 was confirmed by RT-PCR. He had hypoxia on admission with features of right-sided pleural effusion. CT thorax confirmed right-sided pleural effusion with patchy consolidation suggestive of COVID-19 bronchopneumonia in left lung (Figs. 22 and 23). Pleural aspiration yielded frank pus.

Pleural effusion in a patient with COVID-19 is most often due to other causes rather than directly due to Covid. Hence, further evaluation should be done in all patients with COVID-19 with pleural effusion. Causes for pleural effusion in a COVID-19 patient include empyema, hydrothorax due to liver/cardiac/renal disease, tubercular effusion, volume overload or pulmonary embolism [3–5].

#### Learning point

Pleural effusion in a patient with COVID-19 requires detailed evaluation to find the etiology of effusion.

### Case 11

**Fig. 24:**
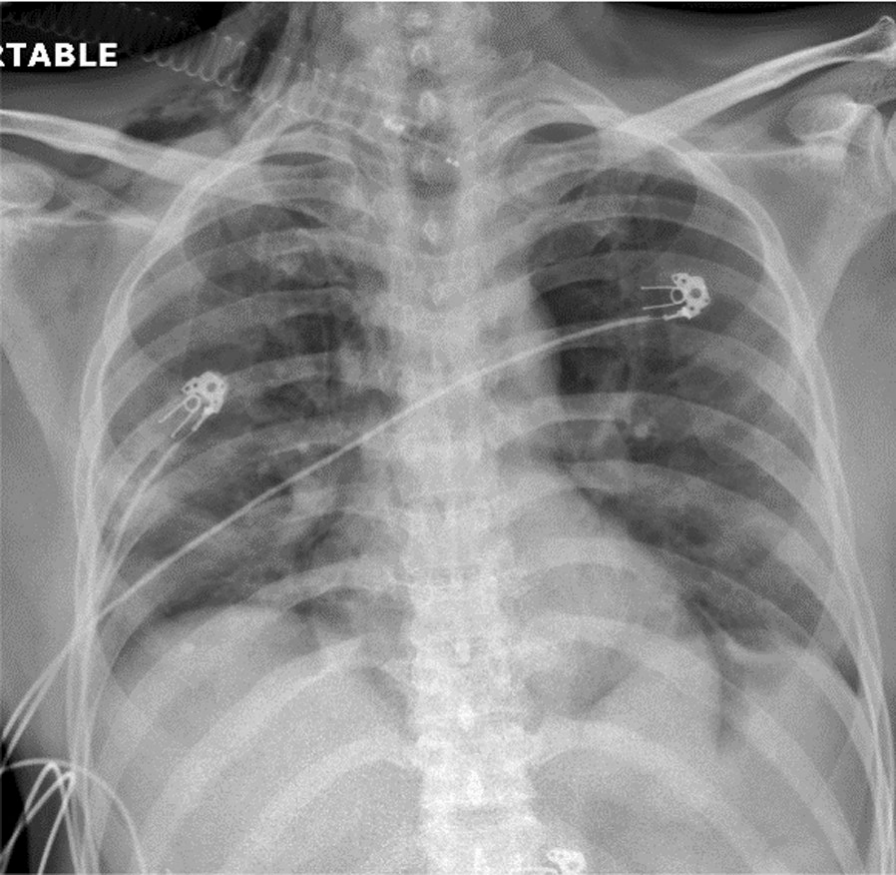
Chest x ray showing mediastinal and surgical emphysema

**Fig. 25:**
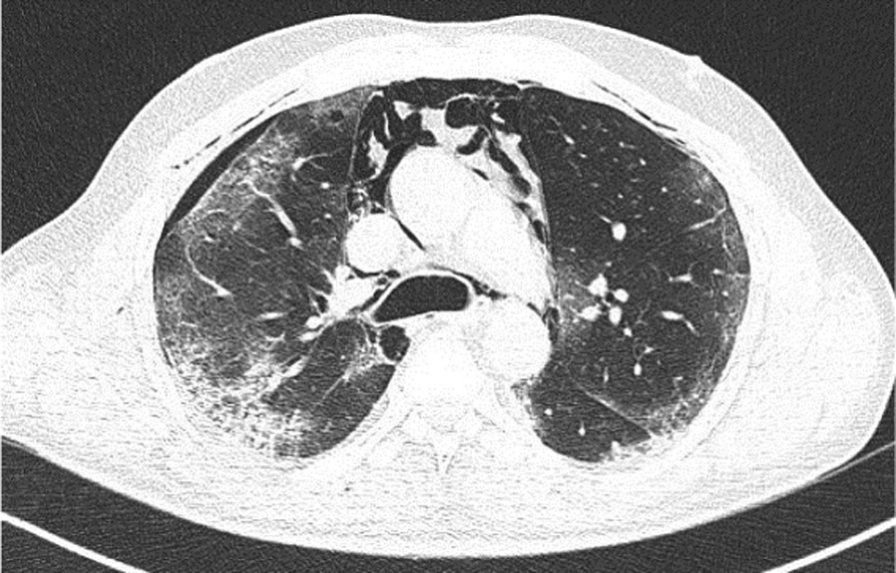
CT scan chest showing mediastinal and surgical emphysema

A 36-year-old male with severe COVID-19 bronchopneumonia on noninvasive ventilation deteriorated suddenly. Clinical examination showed surgical emphysema. Chest X-ray (Fig. 24) showed features of surgical and mediastinal emphysema which was confirmed by CT scan thorax (Fig. 25).

Barotrauma can lead to sudden deterioration and hypoxia in patients with COVID-19 bronchopneumonia. Clinical examination, chest X-ray, and CT scan thorax may help to identify the cause and severity of the complication. The presence of pneumothorax in such cases will require prompt intercostal tube drainage. Barotrauma is more common in patients with moderate or severe disease, those who required oxygen/noninvasive or invasive ventilatory support and prolonged intensive care unit stay [7, 8].

#### Learning point

Barotrauma should be identified and treated promptly as it can lead to sudden deterioration in patients with COVID-19 bronchopneumonia.

### Case 12

**Fig. 26:**
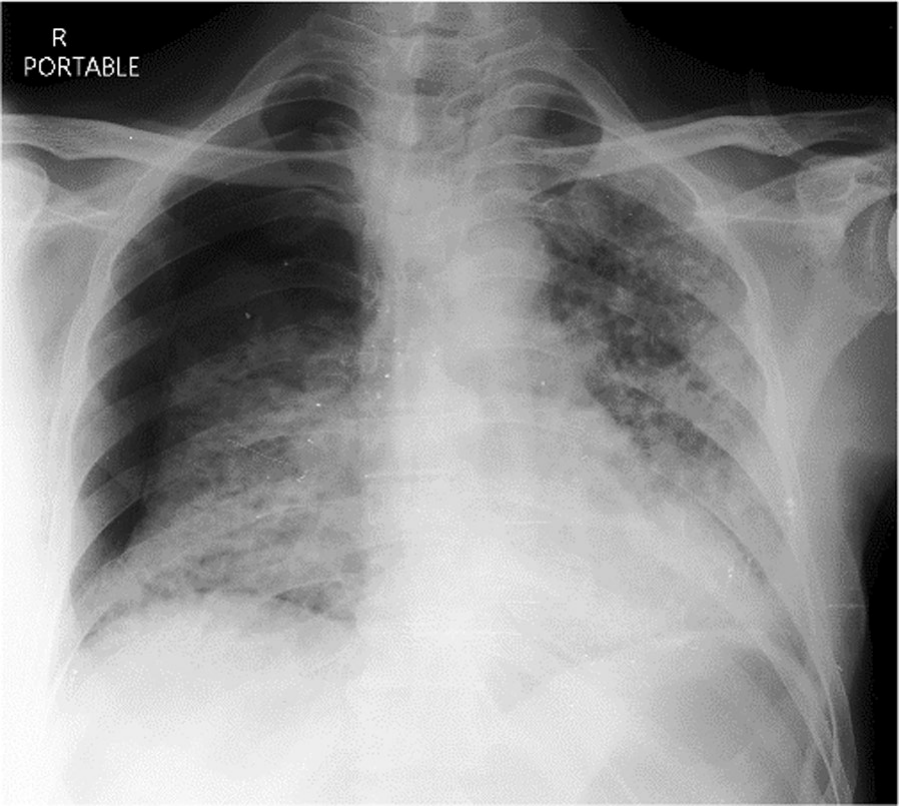
Pneumothorax on right side

A 46-year-old male with severe COVID-19 bronchopneumonia on noninvasive ventilation deteriorated suddenly. Clinical examination showed pneumothorax on right side which was confirmed by chest X-ray (Fig. 26). He was treated with intercostal tube drain.

#### Learning point

Pneumothorax can lead to sudden deterioration in patients with COVID-19 bronchopneumonia. Prompt diagnosis and intercostal tube drain is essential in such patients.

## Conclusions

Awareness about the atypical radiological lesions is essential to avoid misdiagnosis/delayed diagnosis. Some of the atypical radiological features require further evaluation/follow-up and management.

## Data Availability

Available in A J Institute of medical sciences & Research centre, Kuntikana, Mangalore.

## Abbreviations

RT-PCR: Reverse transcription polymerase chain reaction
CT: Computed tomography
